# Molecular Testing in Gliomas: What is Necessary in Routine Clinical Practice?

**DOI:** 10.1007/s11912-024-01602-w

**Published:** 2024-10-03

**Authors:** Iyad Alnahhas

**Affiliations:** Department of Neurology and Neurosurgery, Thomas Jefferson University, 901 Walnut St, Room 310G, Philadelphia, PA 19107 USA

**Keywords:** Astrocytoma, Oligodendroglioma, Next-generation sequencing, IDH, EGFR, MGMT

## Abstract

**Purpose of review:**

A number of molecular characteristics are essential for accurate diagnosis and prognostication in glioma.

**Recent findings:**

The 2021 WHO classification of brain tumors and recent Food and Drug Administration (FDA) pathology agnostic drug approvals highlight the importance of molecular testing in the management of glioma.

**Summary:**

For diffuse gliomas, it is important to identify IDH mutations, given the favorable clinical behavior and potential for using FDA approved IDH inhibitors in the near future. MGMT promoter methylation testing is the most established molecular marker for response to temozolomide in IDH wild-type glioblastoma and in turn impacts overall survival. Moreover, identification of certain mutations and molecular markers, such as BRAF V600E, hypermutation or elevated tumor-mutational burden and NTRK fusions allow for the use of FDA approved agents that are tumor-agnostic. Finally, molecular testing opens options for clinical trials that are essential for diseases with limited treatment options like gliomas.

## Introduction

Diffuse gliomas are the most common malignant primary brain tumors in adults [1]. Diffuse gliomas are thought to arise from glial precursor (progenitor) cells. They include astrocytoma, oligodendroglioma and ependymoma, among a few other rare histopathologies such as pleomorphic xanthoastrocytoma (PXA) [2]. The advances in surgical techniques have allowed sampling and consequently characterization of tumors in deep difficult-to-reach structures in the brain such as the brainstem and identification of mutations such as H3K27M.

Diffuse gliomas remain challenging to treat and continue to carry significant morbidity and mortality. The mainstay treatment modalities for diffuse gliomas include surgery, radiation and chemotherapy, although advanced immunotherapies and targeted therapies are finally starting to show promise against these tumors. In this review, we outline the most relevant molecular markers for diagnosis, prognostication and treatment decisions and clinical practice in diffuse gliomas.

## The Most Common Molecular Alterations in Diffuse Glioma

The advances in DNA sequencing and the application of which to cancer samples started to highlight the prognostic significance of certain molecular alterations in diffuse gliomas. In 2009, a series of seminal studies identified isocitrate dehydrogenase (IDH) mutations in gliomas [3, 4] and they were shown to carry favorable prognosis compared to their IDH wild-type (IDHwt) counterparts. IDH1 R132H mutation is the most common IDH mutation in gliomas for which there is an immunohistochemistry (IHC) antibody available. However, there are other non-canonical IDH1 and IDH2 mutations. The IDH enzyme is key in Krebs cycle and IDH mutations lead to metabolic and epigenetic reprogramming that ultimately lead to gliomagenesis [5]. Similarly, the combined deletion of chromosomes 1p and 19q was identified to carry favorable prognosis in gliomas [6]. Eventually, 1p19q co-deletion, along with the presence of an IDH mutation, became pathognomonic for oligodendrogliomas that carry the best prognosis among diffuse gliomas in adults. Patients with oligodendrogliomas were found to benefit significantly from adding chemotherapy (procarbazine, lomustine and vincristine) to radiotherapy [7, 8].

Next-generation sequencing (NGS) advances eventually paved the way for The Cancer Genome Atlas Project (TCGA). Glioblastoma (GBM) was among the first cancers to be profiled by TCGA [9]. TCGA generated detailed information on the genomic and epigenomic alterations in GBM [9]. At the chromosomal level, chromosome 7 gain (containing EGFR) and chromosome 10 loss (containing PTEN among other tumor suppressor genes) are thought to be early initiating events in the gliomagenesis of IDHwt GBM [10]. At the DNA level, the most common alterations involve the receptor tyrosine kinase pathway (e.g., amplification of the epidermal growth factor receptor (EGFR), phosphatidylinositol 3-kinase pathway (e.g., deletion of the immunosuppressor gene phosphatase and tensin homolog (PTEN), cell cycle pathway (eg, mutations and deletions in CDKN2A/B), p53 pathway, and telomere length maintaining pathways (e.g., TERT promoter mutations). Unsupervised hierarchical clustering of gene expression data from the TCGA network recognized 4 distinct molecular GBM subtypes: proneural, neural, classical, and mesenchymal [11]. This was later specified to proneural, classical, and mesenchymal in IDHwt GBM. The proneural subtype was characterized by abnormalities in platelet-derived growth factor receptor (PDGFR), whereas the classical and mesenchymal subtypes were characterized by EGFR and NF1 mutations, respectively [12].

## The Updated WHO Classification of Brain Tumors

Based on all the above, the 2016 World Health Organization (WHO) Classification of Tumors of the Central Nervous System was a significant update over the 2007 fourth edition. For the first time, the WHO classification used molecular characterization in addition to histology to define tumor entities. The fifth edition of the WHO Classification of Tumors of the Central Nervous System (CNS) was published in 2021 [2] and built on the fourth edition published in 2016. It utilized recommendations from the Consortium to Inform Molecular and Practical Approaches to CNS Tumor Taxonomy (cIMPACT-NOW). cIMPACT-NOW issues frequently published reports to keep up with new evidence and data between the more spread out WHO classification updates.

Per the 2021 WHO classification [2], adult-type diffuse gliomas include IDH-mutant astrocytoma (grade II-IV), 1p19q co-deleted oligodendroglioma (grade II or III) and IDHwt astrocytoma (grade II-IV). The term glioblastoma is reserved for IDHwt astrocytoma grade IV. H3 K27M-altered diffuse midline glioma and H3 G34-mutant diffuse hemispheric glioma are classified under IDHwt pediatric-type diffuse high-grade gliomas, but they are entities seen in adults as well. Ependymomas are classified into supratentorial ZFTA fusion-positive, supratentorial YAP1-fusion positive, posterior fossa ependymoma group A and posterior fossa ependymoma group B.

## The cIMPACT-NOW Updates

Per the cIMPACT-NOW update 1 [13], the qualifier NOS (Not Otherwise Specified) should be used when there is insufficient molecular information to classify a tumor under one of the entities above. Similarly, the qualifier NEC (Not Elsewhere Classified) should be used when the molecular analysis fails to identify any molecular alterations that would render the diffuse glioma fit under one of the entities above.

The cIMPACT-NOW update 2 [14] specified that the the diagnosis of IDH-mutant astrocytoma can be rendered in the absence of 1p19q testing in case of loss of ATRX nuclear expression on IHC and/or strong, diffuse p54 immunopositivity. This is important in situations where testing for 1p19q co-deletion is not available.

Very importantly, the cIMPACT-NOW update 3 [15] determined that there is sufficient evidence that diffuse IDHwt astrocytomas grade II or III that have specific molecular characteristics follow aggressive clinical behavior similar to IDHwt GBM and should be considered as such. These molecular characteristics include EGFR amplification, combined whole chromosome 7 gain and whole chromosome 10 loss (+ 7/-10) and/or TERT promoter mutation.

Mirroring the above in IDH-mutant diffuse astrocytoma, the cIMPAT-NOW update 5 [16] highlighted the homozygous deletion of CDKN2A/B as a marker for poor prognosis in these tumors. As such, the presence of CDKN2A/B homozygous deletion in an IDH-mutant astrocytoma of any grade upgrades the tumor to a grade IV.

## The Molecular Markers in Glioma with Therapeutic Implications

### # IDH mutations

The identification of IDH mutations in diffuse gliomas is important not only for accurate diagnosis but also for treatment decisions. The IDH1/2 inhibitor vorasidenib was recently shown in a double-blind, placebo-controlled phase 3 clinical trial to prolong PFS (27.7 months vs. 11.1 months) and time-to-next intervention in patients with IDH-mutant grade II astrocytomas and oligoendrogliomas [17]. This is of utmost important as patients diagnosed with IDH-mutant grade II gliomas are often younger and delaying their need for radiation and/or chemotherapy leads to improvement in their quality of life and decrease in morbidity, especially given the well-tolerated side effect profile of IDH inhibitors. The Food and Drug Administration (FDA) approved vorasidenib for grade II IDH-mutant astrocytoma or oligodendroglioma in August 2024. Moreover, the field anticipates soon-to-open clinical trials that will test vorasidenib as an adjunct treatment to radiation and chemotherapy for grade III gliomas- especially in the case of non-enhancing disease. The results of these trials might expand the FDA approval indications of vorasidenib. To this point, the National Comprehensive Cancer Network (NCCN) guidelines list ivosidenib – an IDH1 inhibitor- as a treatment option for recurrent IDH1 mutant grade II gliomas and in certain circumstances grade III and IV gliomas (Referenced with permission from the NCCN Clinical Practice Guidelines in Oncology (NCCN Guidelines®) for Guideline Name V.2.2024. © National Comprehensive Cancer Network, Inc. 2024. All rights reserved. Accessed [Aug 30, 2024].

### # MGMT Promoter Methylation

The standard of care for IDHwt GBM includes maximal safe resection followed by concurrent radiotherapy with an oral alkylating agent (temozolomide) and adjuvant temozolomide [18]. The promoter methylation status of O-6-methylguanine-DNA methyltransferase (MGMTp) is the most established molecular predictive marker for response to temozolomide and accordingly impacts overall survival in GBM [19]. Previous literature suggests that the median overall survival (OS) for patients with unmethylated MGMTp GBM is 14.11 months with a median progression-free survival (PFS) of 4.99 months. In contrast, the median OS for patients with methylated MGMTp GBM is 24.59 months with a PFS of 9.51 months [20]. Therefore, knowing the MGMTp methylation status aids in interpreting the brain MRIs during the patients’ treatment course. Pseudoprogression and treatment-related changes—that often look alike tumor progression on MRI- are more common in patients with MGMTp methylated GBM [21]. The benefit of temozolomide in MGMTp unmethylated GBM is minimal enough that it is now accepted to drop temozolomide when treating MGMTp unmethylated GBM patients, especially the elderly [22].

There are a few methods for testing for MGMT promoter methylation in clinical practice: direct bisulfate sequencing (dBiSeq), methylation specific high-resolution melting (MS-HRM), methylation-specific polymerase chain reaction (MSP) and pyrosequencing. The latter two methods are the most commonly adopted. Genome-wide methylation profiling using microarrays and NGS technologies also identifies MGMT promoter methylation status, however, this technology is not widely available.

### # BRAF V600E Mutation

In June 2022, the FDA granted accelerated approval to dabrafenib (BRAF inhibitor) in combination with trametinib (MEK inhibitor) for the treatment of adult and pediatric patients ≥ 6 years of age with solid tumors with BRAF V600E mutations who have progressed following prior treatment. This is agnostic of tumor pathology and as such applies to gliomas. BRAF V600 mutations – leading to mitogen-activated protein kinase (MAPK) pathway activation – can be found in 5–15% of low-grade gliomas, including PXA (60–80%), gangliogliomas (20–70%), pilocytic astrocytomas (10%), and less frequently in GBM (approximately 3%) [23]. The combination dabrafenib with trametinib was studied in the phase II Rare Oncology Agnostic Research (ROAR) basket trial. 45 patients with low-grade and high-grade gliomas were included and response rates ranged from 69% in the low-grade glioma cohort and 33% in the high-grade glioma cohort. The FDA approval allows clinicians to utilize this treatment regimen, especially in diseases with limited treatment options such as gliomas.

### # Tumor Mutational Burden

Similarly, in June 2020, the FDA granted accelerated approval to pembrolizumab for the treatment of adult and pediatric patients with solid tumors that have tumor mutational burden-high (TMB-H) (defined as ≥ 10 mutations/megabase (mut/Mb). Gliomas typically have a low TMB and a highly immunosuppressive microenvironment. This in part explains the unfortunate negative results of two large phase III clinical trials (Checkmate 548 and Checkmate 498) in patients with newly diagnosed IDHwt GBM with MGMTp methylated and unmethylated disease, respectively [24, 25]. The addition of nivolumab (a checkpoint inhibitor of PD-1) to radiation with or without temozolomide failed to improve survival. PD-L1 expression did not affect survival in either study. TMB-H or hypermutation is detected in high-grade diffuse gliomas, especially in the recurrent setting after treatment with the alkylating agent temozolomide (16.6% versus 2.0% in newly diagnosed tumors) [26]. However, despite the availability of the testing and the FDA approved indication, hypermutation in gliomas tends to be subclonal and does not generate optimal anti-tumor responses, and therefore the response rates to checkpoint blockade may still be limited [26]. So, despite the fact that hypermutation may occur in the recurrent setting, in the Checkmate 143 trial, median OS was comparable between nivolumab and bevacizumab in recurrent GBM [27].

### # Neurotrophic Receptor Tyrosine Kinase (NTRK) Fusions

The FDA has approved three NTRK inhibtors: repotrectinib, entrectinib and larotrectinib, for for adult and pediatric patients with solid tumors that have NTRK fusions. Even though NTRK fusions are found in only 1–2% of GBM [28], response rates have been recorded in up to 30% of high-grade and low-grade glioma patients with larotrectenib [29].

### # EGFR Amplification/ Gain-of-Function Mutations

EGFR alterations (amplification and gain-of-function mutations) are quite common and occur in about half of the time in IDHwt GBM [9]. EGFR inhibitors have had numerous clinical successes in non-small cell lung cancer (NSCLC). However, EGFR inhibition has proven disappointing over the years in GBM. Early studies with first- and second-generation tyrosine kinase inhibitors (TKIs) were followed by large phase III clinical trials with vaccines and monoclonal antibodies against EGFR [30, 31]. However, all these trials failed to improve survival in GBM. This speaks to the heterogeneity of the GBM and is in part explained by multiple receptor tyrosine kinase (RTK) activation [32]. However, efforts to target EGFR have continued in GBM including recent efforts utilizing engineered chimeric antigen receptor (CAR) T-cells [33, 34]. However, even these small phase I studies show limited efficacy and considerable toxicity from this treatment. Testing for EGFR alterations may still open clinical trial opportunities for patients, however Table 1.

**Table 1:** highlights the FDA approved targeted therapies and immunotherapies for primary brain tumors

FDA approved treatment	Indication
Belzutifan	von Hippel-Lindau (VHL) disease with central nervous system (CNS) hemangioblastomas
Bevacizumab	Recurrent GBM
Dabrafenib and Trametinib	Solid tumors with BRAF V600E mutation
Everolimus	Tuberous sclerosis complex-associated partial-onset seizures
Tovorafenib	Pediatric low-grade glioma with a BRAF alteration
Vorasidenib	IDH mutant grade II astrocytoma and oligodendroglioma
Pembrolizumab	Tumor mutational burden-high solid tumors

Given the above described importance of molecular testing for accurate cancer classification and treatment decisions, it is disappointing when some insurance companies – still in this day and age – deny coverage and reimbursements to molecular testing, reinforcing inequity in percision medicine [35].

## Conclusions

The molecular characterization of tumors has yielded valuable insights toward accurate identification as well as understanding of the tumor behavior and prognosis. It also gave birth to targeted therapies. Despite the challenges in the treatment of GBM and the failure of many clinical trials using targeted therapy and immunotherapy, there remains importance in identifying a number of molecular markers. Namely, it is essential to differentiate between IDH-mutant and IDHwt astrocytoma, given the vastly different clinical behavior, and the potential for using FDA approved IDH inhibitors in the near future. Similarly, academic neuropathology practices only diagnose an oligodendroglioma if an IDH mutation and 1p19q co-deletion are confirmed. The analysis of MGMTp methylation status is essential for prognostication in IDHwt GBM. The benefit of temozolomide in MGMTp unmethylated GBM is minimal enough that it is now accepted for new clinical trials to drop temozolomide from the experimental arms in MGMTp unmethylated GBM patients.

Moreover, NGS of glioma is now justified to look for CDKN2A/B homozygous deletions that upgrade IDH-mutant astrocytomas to grade IV, and EGFR amplification, chromosome (+ 7/-10) and TERT promoter mutations that upgrade IDHwt astrocytomas to grade IV. NGS also allows to identify BRAF V600E mutations and TMB-H tumors as well as other rare molecular alterations (such as NTRK fusions) that have FDA approved tumor-agnostic treatment options.

## Key References

- Louis DN, Perry A, Wesseling P, Brat DJ, Cree IA, Figarella-Branger D, et al. The 2021 WHO Classification of Tumors of the Central Nervous System: a summary. Neuro Oncol. 2021;23(8):1231–51.
  - The WHO 2021 classification of brain tumors summarizes the molecular characteristics of most clinical relevance in gliomas and other brain tumors.
- Mellinghoff IK, van den Bent MJ, Blumenthal DT, Touat M, Peters KB, Clarke J, et al. Vorasidenib in IDH1- or IDH2-Mutant Low-Grade Glioma. N Engl J Med. 2023;389(7):589–601.
  - Vorasidenib will likely be the first IDH inhibitor to be FDA approved in IDH mutant glioma.
- Wen PY, Stein A, van den Bent M, De Greve J, Wick A, de Vos F, et al. Dabrafenib plus trametinib in patients with BRAF(V600E)-mutant low-grade and high-grade glioma (ROAR): a multicentre, open-label, single-arm, phase 2, basket trial. Lancet Oncol. 2022;23(1):53–64.
  - There is a tumor-agnostic FDA approved indication for dabrafenib and trametinib in any progressive solid tumor that harbors the BRAF V600E mutation.

## Data Availability

No datasets were generated or analysed during the current study.
